# Titanium-based nanostructures for the photocatalytic degradation of antibiotics

**DOI:** 10.1039/d6ra04232e

**Published:** 2026-08-17

**Authors:** Ana-Maria Fulgheci, Denisa Ficai, Anton Ficai

**Affiliations:** a Faculty of Chemical Engineering and Biotechnologies, National University of Science and Technology Politehnica Bucharest 1-7 Polizu St. 011061 Bucharest Romania anton.ficai@upb.ro; b Control Pollution Department, National Research and Development Institute for Industrial Ecology—ECOIND Drumul Podu Dambovitei 57-73 060652 Bucharest Romania; c Academy of Romanian Scientists Ilfov Street 3 050044 Bucharest Romania

## Abstract

The use of pharmaceutical compounds, mainly antibiotics, for human and veterinary applications has increased in recent years. The human and animal body only partially metabolizes administered antibiotics, and the remaining drugs, whether active or unchanged, are eliminated into the environment through urine and feces. Antibiotic-polluted wastewater is treated at wastewater treatment plants (WWTPs), although they cannot completely remove antibiotics. Since biological treatments are insufficient to break down the majority of antibiotics, more effective ways to degrade them into CO_2_ and H_2_O must be developed. Advanced oxidation processes (AOPs) are some of the best solutions. Photocatalysis offers many advantages over other advanced oxidation technologies. In recent years, many Ti-based nanostructures have been developed for the photocatalytic removal of antibiotics from the environment. This article summarizes the most recent studies on titanium-based nanocomposites and how they can be used to photodegrade antibiotics in water. The study also considers the safety of these techniques by evaluating the degradation products that are formed and their potential to induce resistance.

## Abstract 2

This work presents an overview of existing photocatalysts based on titanium-based nanostructures. New opportunities and future perspectives are presented to assure the safer degradation of antibiotics.

## Graphical abstract

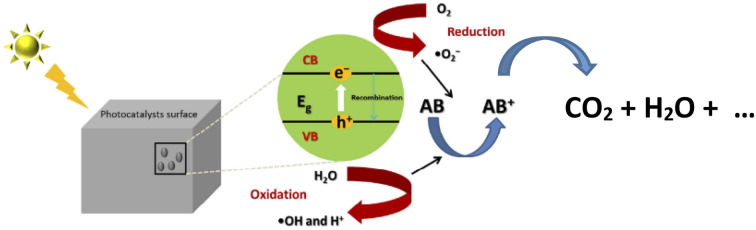

## 1. Introduction

Antibiotics, substances that destroy, inhibit or reduce the growth and proliferation of microorganisms, infiltrate wastewater treatment systems and eventually make their way into the aquatic environment.^1,2^ Following the discovery of multiple antibiotic-resistant bacteria and antibiotic-resistant genes in surface water, drinking water, and municipal solid waste emissions, it is proved that even extremely low antibiotic levels may encourage the development of antibiotic resistance.^3^

**Fig. 1:**
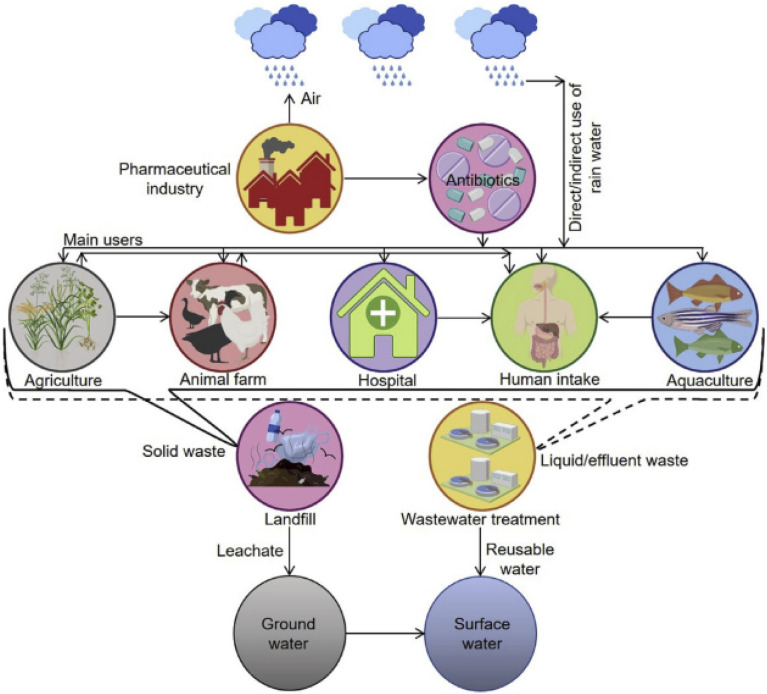
Antibiotic contamination of surface and ground water systems through direct or indirect routes. Reproduced from ref. 1, with permission from Elsevier.

The use of pharmaceutical compounds, primarily antibiotics, for both human and veterinary applications has steadily increased in recent years.^4^ Klein *et al.* examined patterns in antibiotic usage during the period of 2016–2023, paying particular attention to variations in antibiotic consumption with respect to the World Bank income classification.^5^ Between 2016 and 2023, the overall amount of antibiotics consumed increased by 16.3%, from 29.5 to 34.3 billion defined daily doses, and the rate of antibiotic consumption in these nations increased by 10.6%, from 13.7 to 15.2 defined daily doses per 1000 people per day.^5^ The human body only partially metabolizes administered antibiotics, and the remaining drugs, whether altered (metabolites) or unaltered (the antibiotic itself), are released into the environment through urine and feces. Wastewater treatment plants (WWTPs) are able to receive these effluents, but unfortunately, traditional WWTPs cannot (completely) remove antibiotics and their metabolites.^1^ Antibiotics may be present in the sludge and final effluent from wastewater treatment plants. The effluent is released into surface water, and the sludge may be utilized as a fertilizer, and thus, they can disseminate these pollutants and generate antibiotic resistance (Fig. 1).^6^

When antibiotic residues enter the environment, they can harm animal and human health and the biota by spreading antibiotic resistance or contaminating food.^6^ Depending on the type of antibiotic and the organism it affects, different antibiotics have different levels of toxicity. Species that belong to a lower trophic level (algae, cyanobacteria, *etc.*) are more sensitive to antibiotics than organisms that belong to higher trophic levels (fish, crustaceans, *etc.*). Among the classes of antibiotics, macrolides exhibit the highest toxicity toward cyanobacteria and algae, with the EC_50_ (half maximal effective concentration) value being <1 mg L^−1^. The continuous and uncontrolled discharge of antibiotic residues into the environment induces numerous negative effects in aquatic organisms, leading to an increase in the concentration of antibiotic residues in the water (currently, the concentration is in the range of ng L^−1^ or µg L^−1^).^7^ Unfortunately, these negative effects are further potentiated by many other factors, including, but not limited to, pesticides,^8^ heavy metals,^9^ microplastics,^10^ and biocides.^11^

Table 1 lists the main antibiotics detected in hospital effluents and their adverse effects. It is important to mention that the concentration of these antibiotics is strongly correlated with the specificity of the hospital, the treatment schemes they usually adopt, the degradation profile of the antibiotics, their water solubility and adsorption on the sludge, the temperature, *etc.*

**Table 1:** Upper limit of antibiotics detected in hospital effluents and their side effects^12,13^

Compound	Therapeutic group	Maximal content detected (mg L^−1^)	Side effects
Ciprofloxacin	Antibiotics	0.125	Tendonitis and Achilles tendon rupture
			Increased risk of abdominal aorta rupture
			Persistent peripheral neuropathy
Clarithromycin	Antibiotics	0.003	*Clostridium difficile* infection
			Irregular heart rhythms
Doxycycline	Antibiotics	0.007	Hypersensitivity reaction
			Serum sickness-like reaction
			*Clostridium difficile* infection
Erythromycin	Antibiotics	0.083	*Clostridium difficile* infection
			Irregular heart rhythms
Metronidazole	Antibiotics	0.090	Carcinogenic effects in rats and mice
			Peripheral neuropathy
Norfloxacin	Antibiotics	0.044	Tendonitis and Achilles tendon rupture
			Increased risk of abdominal aorta rupture
Penicillin	Antibiotics	0.005	Renal inflammation associated with *Clostridium difficile* infection
Sulfamethoxazole	Antibiotics	0.083	Serum sickness-like syndrome
			Interstitial nephritis
			Lupus-like syndrome
Tetracycline	Antibiotics	0.004	Hypersensitivity reaction
			Serum sickness-like reaction
			*Clostridium difficile* infection
Trimethoprim	Antibiotics	0.015	Serum sickness-like syndrome
			Interstitial nephritis
			Lupus-like syndrome

The inexpensiveness of antibiotics and the excessive use of broad-spectrum antibiotics (for both human and veterinary uses) as a preventative measure are significant contributors to the development of antibiotic resistance.^3^ The high antibiotic usage is reflected in the large fraction of unmetabolized antibiotics that end up in the environment. Environmental resources are further strained by the discharge of the effluent from pharmaceutical factories and wastewater treatment plants. The excessive contamination of the environment with antibiotics encourages the growth and spread of antibiotic-resistant bacteria. Due to their repeated exposure to low antibiotic doses, these bacteria are able to survive, adapt and evolve.^14^ When bacteria in wastewater are attacked by antibiotics, they employ various defense strategies and become resistant to either a specific antibiotic or multiple antibiotics. These microorganisms are referred to as multidrug-resistant bacteria.^15^

The most significant factor in the development of antibiotic resistance seems to be mutations brought on by low antibiotic doses. Even in nonlethal doses, antibiotic exposure can cause some specific mutations in bacteria. The accumulation of these mutations over time can lead to the production of bacterial species that are resistant to antibiotics, making treatment more difficult.^14^ Nguyen *et al.* evaluated antibiotic resistance and biofilm formation^16^ by considering a wide range of antibiotics and bacterial strains (including resistant and clinical strains). It was observed that the minimal inhibitory concentration (MIC) of these antibiotics (Table 1) ranged from tens to thousands of µg L^−1^, which means that bacterial strains exposed to these solutions for hours or few days could develop resistance. This was also proven by Gullberg *et al.*^17^ based on their *in vitro* experiments; they proved that when exposed to even very low antibiotic concentrations (hundreds of times below that of the MIC), bacteria developed antibiotic-resistant genes.

It is vital to put in place initiatives that educate the public about the risks of inappropriate antibiotic usage in order to stop bacteria from becoming increasingly resistant to more and more antibiotics. TiO_2_ is considered a standard photocatalyst due to its properties, such as chemical stability in aqueous media, wide availability, affordability and biocompatibility.^18^ Other nanomaterials used for the photodegradation of pollutants, like ZnO, are susceptible to corrosion and dissolution in basic or acidic media and show faster e^−^/h^+^ pair recombination, while WO_3_ experiences photocorrosion after prolonged exposure to light and has a very narrow absorption edge that prevents it from using photons from the majority of the solar spectrum.^19,20^ α-Fe_2_O_3_ has poor conductivity, a high recombination rate of e^−^/h^+^ pair and low diffusion lengths of holes.^21^ Hence, although TiO_2_ is not as photocatalytically active as these nanomaterials, it is the most stable material, and the results it produces can be considered reproducible. In this review, the latest titanium-based nanostructures used for the photodegradation of antibiotics are presented, as well as how antibiotic degradation byproducts can induce antibiotic resistance. Unfortunately, even though photocatalysis is being utilized for quite a long time, the exact mechanisms of degradation and individual toxicity of the degradation products are not known because the photodegradation process does not always lead to carbon dioxide and water. In addition to other studies in this field, this review includes information about photocatalyst reusability as well as some data regarding the toxicity of the degradation products.

## 2. Biological treatment for antibiotic removal

When antibiotic-contaminated wastewater reaches wastewater treatment plants, biological treatment processes (biodegradation) are insufficiently effective in removing harmful compounds. Considering this, a more complex treatment is needed, which mainly combines biological treatment with other treatment techniques. After being treated with activated sludge, many antibiotics are either not broken down at all or are broken down only partially, and the secondary compounds that are produced as a consequence of the degradation process may persist in the wastewater treatment plant's effluent and have an undetermined level of toxicity.^22^ Each antibiotic's level of biodegradation is determined by its physicochemical properties and chemical structure. In the presence of activated sludge, antibiotics belonging to the sulfonamide class can readily biodegrade; however, the toxicity of the byproducts is not well understood.^23–26^ Unlike sulfonamides, trimethoprim is not easily broken down in the presence of activated sludge under aerobic conditions.^24,26–28^ Aminoglycosides, such as streptomycin, cannot be biodegraded by activated sludge or under aerobic or anaerobic conditions. Through the tests conducted, it has been found that although a part of the antibiotic is adsorbed on the surface of the sludge, its structure remains intact.^26,29–31^ Similar to aminoglycosides, tetracyclines do not biodegrade under aerobic and anaerobic conditions. Biological transformation is not a significant factor in the case of these antibiotics.^26,32^ Penicillin G, one of the β-lactam antibiotics, could undergo biodegradation in both the Zahn–Wellens and closed bottle tests. However, there is not enough information available to assess the level of biodegradation of the other antibiotics in this class.^29,30,33,34^ Since macrolide antibiotics like clarithromycin, azithromycin, and erythromycin are commonly found in European waters, they are listed as emerging pollutants. Following biodegradation experiments, it was discovered that because of the smaller amount of inoculum used, erythromycin cannot be biodegraded through the closed bottle test. Although the Zahn–Wellens test yields superior results, it has been found that the substance shows an inhibitory effect against the microorganisms from the activated sludge.^29,30,34^ The activated sludge microbial community needs to acclimate in order to assess the level of biodegradability of fluoroquinolones using the Zahn–Wellens test or the closed bottle test. Without this pretreatment, fluoroquinolones are resistant to biodegradation.^29,33,34^ The efficiency of this complex process is strongly dependent on the nature of the antibiotic and the treatment conditions.

## 3. Photocatalysis for the removal of antibiotics

Since biological treatments are insufficient to break down the majority of antibiotics, more effective ways to degrade them into CO_2_ and H_2_O must be found. Advanced oxidation processes (AOPs) are some of the best solutions. Reactive oxygen species such as hydroxyl radicals, hydrogen peroxide, ozone and superoxide radical anions are produced *in situ* during these processes. These reactive species possess a strong potential to mineralize contaminants, often breaking them down into CO_2_, H_2_O, organic acids, or various intermediate by-products.^22^

Photocatalysis offers many advantages over other advanced oxidation technologies, including chemical oxidation, biological treatment, ozonation, and adsorption on activated carbon, as comparatively presented in Table 2. High amounts of contaminants can be broken down by chemical oxidation, but the organic load cannot be entirely eliminated by this approach. Other chemical oxidation processes, such as ozonation, also produce secondary oxidative species and demand a lot of energy. Very slow kinetics, the formation of a lot of activated sludge with emerging pollutants that need to be eliminated, and strict temperature and pH control are the drawbacks of biological treatments. Furthermore, activated sludge has a poor rate of degradation of antibiotics. Although adsorption on activated carbon is very effective, it has the drawback of transferring contaminants without breaking them down, which leads to yet another environmental issue.^22^

**Table 2:** Advantages and disadvantages of some technologies used for the removal of antibiotics^22,35,36^

Technology	Advantages	Disadvantages
Biological treatment	Cost-effectiveness and sustainability	Very slow kinetics and poor degradation rates
	Minimization of secondary pollution	High risk of antibiotic resistance
	Effective removal demonstrated in practice	A large amount of activated sludge containing emerging contaminants must be removed
	Versatility *via* co-metabolism	
Activated carbon adsorption	Very effective method	Pollutants are transferred without decomposition
	Low operation costs	High regeneration costs
	Simple operation	
Membrane filtration	Highly effective	Non-destructive process
	No chemicals involved	Unsuitable for large wastewater volumes
		Membrane fouling
		High operation and maintenance costs
Fenton oxidation	Easy and high-degradation operation	The degradation process is influenced by variables such as temperature, pH, H_2_O_2_, and Fe^2+^ concentration
	Possible mineralization of pollutants	This method is limited to acidic conditions
	Iron is non-toxic and abundant	
Ozonation	Environmentally friendly method when combined with biological treatment	Certain products of antibiotic transformation (oxidative by-products) can be very toxic to biological activity
	Efficient method for the decomposition of antibiotics	High energy requirement
	On-site generation of ozone	
Photocatalysis	Efficient method for organic pollutant decomposition	Various factors such as the structure, surface, amount and size of the catalyst, pH, light intensity, pollutant concentration, reaction temperature, and recombination of photogenerated charge carriers affect the degradation process
	Low catalyst concentrations are required	Laboratory scale
	Cheap and highly effective catalysts can be used	Possible toxicity of by-products
	Sunlight can be used for the activation of the catalyst	
	Photocatalysts can be efficiently recovered and reused	
	Pollutants can be converted into harmless substances like H_2_O and CO_2_	

**Fig. 2:**
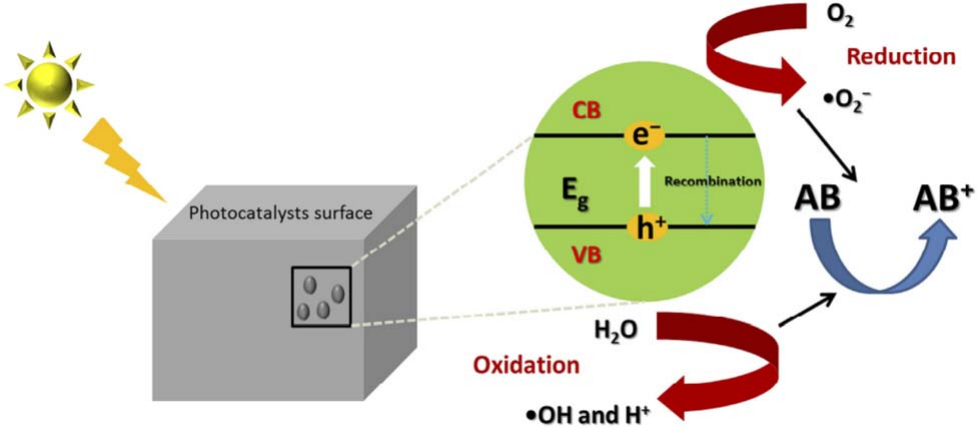
Mechanism of the photocatalytic degradation of the target antibiotic molecule (AB). Reproduced from ref. 37, based on the CC BY license.

Photocatalytic degradation (Fig. 2) consists of three steps: photon absorption, excitation, and reaction. Thus, after the absorption of photons having an energy matching with the band gap of the catalyst, electrons belonging to the valence band are excited and promoted to the conduction band. An electron hole is generated in the valence band. The electrons and electron holes are effectively separated and migrate on the surface of the catalyst, where they participate in secondary reactions with the adsorbed compounds. There is a possibility that the generated electron holes can directly attack toxic compounds, leading to their degradation. Researchers have identified and presented two different kinds of systematic theories regarding degradation pathways. If the conduction band potential of the catalyst is negative compared to the redox potential of the O_2_/˙O_2_^−^ pair, the excited electrons react with electron acceptors, such as O_2_ deposited on the surface of the catalyst or dissolved in water, reducing it to the superoxide radical anion. When the holes move to the photocatalyst surface, a different pathway, known as the oxidative pathway, is initiated. Depending on the medium's acidity or alkalinity, this process results in the formation of hydroxyl radicals when H_2_O/OH^−^ is oxidized. After excitation, hydrogen ions may recombine with the electrons to produce energy, reducing the photodegradation efficiency. It is observed that in this scenario, the standard redox potential of the photocatalyst needs to be greater than that of ˙OH/OH^−^. In the photocatalytic process, both reactive radicals, ˙OH and ˙O_2_^−^, are extremely potent oxidizing agents.^37,38^

The wavelength and intensity of the applied light have a significant impact on the photocatalytic degradation of antibiotics. The quantity of photons in a reaction system increases as the light intensity rises. As a result, additional charge carriers are produced, which participate in photocatalytic oxidation and increase the rate at which antibiotics are removed. Pure TiO_2_ can only use light in the UV region (wavelengths below 390 nm) because of its band gaps of 3.0 eV for the rutile phase and 3.2 eV for the anatase phase.^36^ Rapid electron–hole recombination and poor interaction with hydrophobic organic contaminants are two additional challenges.^39^ Electron mobility can be influenced by the structure of the material. For example, TiO_2_ nanoparticles have a lower electron mobility than highly ordered TiO_2_ nanotubes.^40^ Elemental doping (with metals and nonmetals), heterojunction design, defect engineering and the use of surface plasmon resonance effects are just a few of the techniques that have been developed to overcome these limitations. These techniques modify TiO_2_'s electronic structure to enhance carrier transport and charge separation, lower its band gap, and boost absorption of the visible wavelength.^41–45^

Metal doping is the process of adding metal ions to the TiO_2_ crystal lattice, either interstitially or substitutionally (replacing Ti^4+^ ions). Visible light absorption and interband electronic transitions can be facilitated by the introduction of energy levels in the mid-band by metal dopants. Furthermore, some metal ions like Fe and Ag promote charge separation and the production of reactive oxygen species, in addition to improving the absorption of visible light.^41^ In non-metal doping, light elements like nitrogen, carbon, sulfur, boron, and fluorine are incorporated into the TiO_2_ lattice, frequently in place of lattice oxygen. By adding impurity states or hybridizing their p orbitals with O 2p states, these dopants alter the valence band structure, lowering the band gap and permitting activation by visible light. One of the most studied non-metal doping strategies is nitrogen doping.^41^

Defect engineering is the deliberate modification of crystalline imperfections in the lattice of TiO_2_ to tailor its optical absorption properties, electronic structure, and photoelectrocatalytic activity. Because of their significant effects on charge carrier dynamics and visible light absorption, oxygen vacancies have garnered the most attention among all defect types.^41^

An efficient method to improve light absorption, inhibit charge recombination, and promote interfacial charge transfer is to create heterojunctions by combining TiO_2_ with other semiconductors. Heterojunctions are categorized into three types, namely, type I (transverse gap), type II (staggered gap), and type III (interrupted gap), based on the relative alignment of the bands of the semiconductor components.^41^

The coherent oscillation of conduction band electrons at the interface between a metallic nanoparticle and a dielectric medium when exposed to light at a resonant frequency is known as surface plasmon resonance (SPR). Through a few mechanisms, such as broadening of the light absorption spectrum into the visible region, local electromagnetic field enhancement, and hot electron injection into the semiconductor, SPR-active nanostructures can greatly enhance the photoelectrocatalytic performance of TiO_2_.^41^

## 4. Ti-based nanostructures for antibiotic photodegradation

**Fig. 3:**
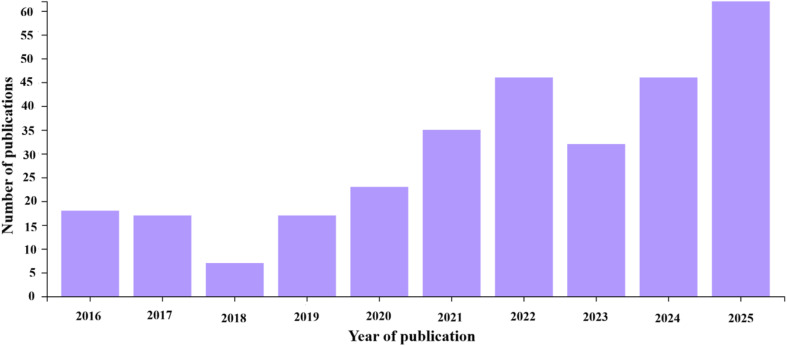
Publications on antibiotic degradation using titanium-based photocatalysts from 2016 to 2025 (the statistics were generated using the Web of Science database; keywords: photocatalysis, titanium, antibiotic, degradation).

In recent years, more and more Ti-based nanostructures have been developed for the photocatalytic removal of antibiotics from the environment (Fig. 3).

Because of its exceptional qualities, including high photocatalytic activity, nontoxicity, chemical stability, the capacity to be coated as a thin film on a substrate, and environmental friendliness, TiO_2_ is gaining the most attention among all semiconductor photocatalysts. There are three distinct polymorphs of TiO_2_: anatase, rutile, and brookite. The most stable form of TiO_2_ is rutile, while anatase is the form with the highest photocatalytic activity. Despite the fact that anatase TiO_2_ has a larger band gap than rutile TiO_2_, anatase TiO_2_ presents an indirect band gap, while rutile TiO_2_ has a direct band gap. Due to its superior lifetime, which inhibits the recombination of electron–electron hole pairs (e^−^/h^+^), anatase TiO_2_ has superior catalytic activity to rutile TiO_2_.^46^

The use of TiO_2_ or other titanium-doped materials as photocatalysts for the degradation of antibiotics has been the subject of numerous studies in the specialized literature.

### 4.1 MCM-41-supported titanium catalysts

Zhou *et al.*^47^ synthesized four Ti-doped Mobil Composition of Matter no. 41 (MCM-41) catalysts with various Si/Ti molar ratios. Additionally, two control samples, TiO_2_ and MCM-41, were synthesized. The synthesized samples were physicochemically characterized using X-ray diffraction (XRD) to identify the crystalline phases of the photocatalysts and transmission electron microscopy (TEM) to analyze the size and shape of the particles; their pore sizes and specific surface areas were examined using N_2_ adsorption/desorption isotherms, and Fourier transform infrared (FTIR) spectroscopy was used to analyze the chemical bond vibrations in the structure of the photocatalysts. Analytical data indicated that doping MCM-41 with Ti changed its lattice parameters. Additionally, there was a slight decrease in the specific surface area compared to MCM-41. It was observed that excessive Ti doping resulted in an overabundance of TiO_2_ particles, which affected the adsorption and photodegradation capacity of the synthesized materials. Through adsorption/desorption analyses, it was found that there was no significant decrease in the adsorption capacity of the materials doped with Ti. In terms of the catalytic activity, it was found that the presence of sulfate and bicarbonate anions in simulated wastewater samples from the hennery reduced the photocatalytic capacity. The catalyst with a molar ratio of Si/Ti of 25 exhibited the highest photocatalytic activity among the four synthesized photocatalysts, breaking down more than 99% of oxytetracycline from the synthetic solution in 150 minutes, with the resulting solution having no antimicrobial activity. The catalytic activity dropped to 98% after the catalyst was reused five times. Through the analysis of the samples subjected to degradation, 10 intermediate degradation products were identified, whose concentrations initially increased and then decreased, which indicated that no new species that could potentially induce biotoxicity were generated.^47^

Chen *et al.*^48^ synthesized Ti-MCM-41 using a microwave-assisted hydrothermal method. Using XRD analysis, the doping of Ti in the MCM-41 structure was confirmed due to the structural changes that were observed compared to pure MCM-41. Brunauer–Emmett–Teller (BET) analysis showed that the synthesized catalyst had a typical mesoporous structure, with a high pore volume and uniformly sized pores. The band corresponding to the Ti–O–Si bond, which was observed at 1628 cm^−1^ in the catalyst's FTIR spectrum, highlighted the Ti doping of MCM-41. UV-Vis analysis showed that the absorption band of the Ti-MCM-41 catalyst considerably shifted to 330 nm, which was advantageous for photocatalytic applications. According to the results of the photodegradation experiments, Ti-MCM-41 degraded oxytetracycline in an acidic medium with the highest efficiency. At pH = 3, the total organic carbon removal effectiveness was 87.0%, whereas at pH = 10, it was 50.9%.^48^ Four degradation products of oxytetracycline were proposed.^48^ According to previous studies, the toxicity of the photolysis byproducts of oxytetracycline, combined with the toxicity of the parent compound, is higher than the toxicity of the original antibiotic solution, resulting in a more aggressive effect on bacteria and aquatic systems.^49^

### 4.2 Pure TiO_2_-based catalysts

Abromaitis *et al.*^50^ prepared TiO_2_ nanotubes by anodic oxidation. Scanning electron microscopy (SEM) analysis highlighted the well-defined structure of the nanotubes with a pore diameter of approximately 67 nm. The layer of titanium dioxide nanotube arrays (TiO_2_ NTAs) deposited on the substrate had a moderate thickness. TiO_2_ NTAs were found to have an average crystallite size of 36.25 nm. XRD analysis revealed that TiO_2_ nanotubes were composed of multiple crystallites, with a major proportion of the anatase phase. A number of AOPs, including photocatalysis, photolysis, catalytic ozonation and ozonation, were investigated in a laboratory reactor to determine their removal capacity for ciprofloxacin (CIP) from the household wastewater treatment plant's secondary effluent and model wastewater. The most efficient technique was found to be the combination of the TiO_2_ nanotube array photocatalyst and the photocatalytic ozonation process, which achieved the highest ciprofloxacin removal rate constants. Furthermore, it took 8 minutes to remove 1 mg per L ciprofloxacin from model wastewater and 15 minutes to remove the same amount from the secondary effluent. The elimination efficiencies of ciprofloxacin were 53–69% and 93–100%, respectively, following photolytic treatment and photocatalytic oxidation, respectively. By contrast, when ozone was added to the photocatalytic ozonation reactor, ciprofloxacin was always completely removed.^50^

**Fig. 4:**
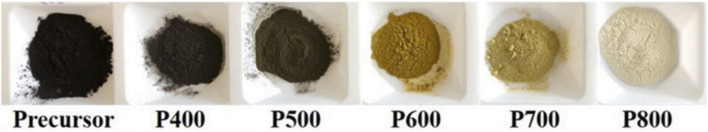
Precursor and samples calcined at different temperatures from 400 °C to 800 °C. Adapted from ref. 51, with permission from Elsevier.

The successful preparation of colored powders with Ti_2_O_3_, Ti_3_O_5_ and TiO_2_ (Fig. 4) was documented by Figueroa-Torres *et al.*^51^ using a straightforward controlled oxidation reaction of a Ti_2_O_3_ precursor from 400 °C to 800 °C. Depending on the oxidation temperature, mixtures of Ti_2_O_3_, Ti_3_O_5_ and TiO_2_ (anatase and rutile) were obtained. The composition of the mixtures is presented in Table 3. The oxidative degradation of ofloxacin under simulated sunlight irradiation was used to assess the samples' photocatalytic performance. After 120 minutes of sunlight exposure, the Ti_2_O_3_ precursor only degraded 15.4% of ofloxacin, demonstrating poor photocatalytic activity. With a 31.7% breakdown of the initial ofloxacin concentration, the P400 sample showed increased photoactivity. The P500 sample exhibited 54.4% degradation, the highest photocatalytic performance. The P600, P700, and P800 samples only achieved 19.6%, 15.6%, and 13.5% antibiotic degradation, respectively, indicating a significant drop in the photocatalytic activity. The commercial material, TiO_2_ P25, showed 100% degradation. P500 exhibited a strong absorption of visible light, which is a crucial feature for real-world applications under natural sunlight, even though TiO_2_ P25 has a higher efficiency than P500. The P500 sample was used to assess the bactericidal effect on *E. coli* and *S. aureus* since it exhibited the highest photocatalytic activity. When the P500 sample was not exposed to radiation, it had no bactericidal effect. When P500 was irradiated for 3 hours, it was found that 38% of *S. aureus* and 49% of *E. coli* cells survived, which was higher than when P25 was used.^51^

**Table 3:** Composition of the precursor and oxidized samples prepared by Figueroa-Torres *et al.*^51^

Oxide type	Sample						
	Precursor	P400	P500	P600	P700	P800	P25
Ti_2_O_3_	80%	83%	22%	6%	—	—	—
Ti_3_O_5_	16%	14%	7%	—	—	—	—
γ-Ti_3_O_5_	4%	3%	—	—	—	—	—
Rutile	—	—	50%	75%	82%	84%	15%
Anatase	—	—	21%	19%	18%	16%	85%

Feng *et al.*^52^ synthesized an aqueous nanodispersion of TiO_2_ that had a high specific surface area and a uniform particle size of 10 nm. Its photocatalytic activity was tested both under visible and UV light irradiation. When the sample was irradiated with UV light, it was found that the best photocatalytic activity was achieved at a catalyst concentration of 40 mg L^−1^ after 30 minutes of irradiation. The synthesized nanodispersion exhibited superior catalytic activity to TiO_2_ P25. In addition, it was found that at acidic pH values of the testing solution, the degradation was inhibited due to a decrease in the number of active species. The results obtained from the visible spectrum highlighted the duality of the photocatalyst, which degraded 93% of the tetracycline solution when 200 mg L^−1^ of the catalyst was used. It was found that increasing the catalyst concentration to 300 mg L^−1^ did not lead to a significant improvement in the degradation rate, which was explained by the increasingly difficult penetration of light radiation as the amount of the catalyst increased. Compared to commercial TiO_2_, the synthesized TiO_2_ nanodispersion showed an increase in the degradation rate to 90% in just 20 minutes of irradiation, while only 63% was achieved by TiO_2_ P25. Furthermore, the reusability rate of the nanodispersion was evaluated, and it was shown that the tetracycline removal percentage remained high at 94% after five testing cycles. Two pathways for tetracycline degradation by the TiO_2_ nanodispersion under UV irradiation were proposed. Although it was assumed that tetracycline would break down into CO_2_, H_2_O, and ammonium salts, the tetracycline solution was not quantitatively analyzed following irradiation to determine the structure of the degradation products.^52^

**Fig. 5:**
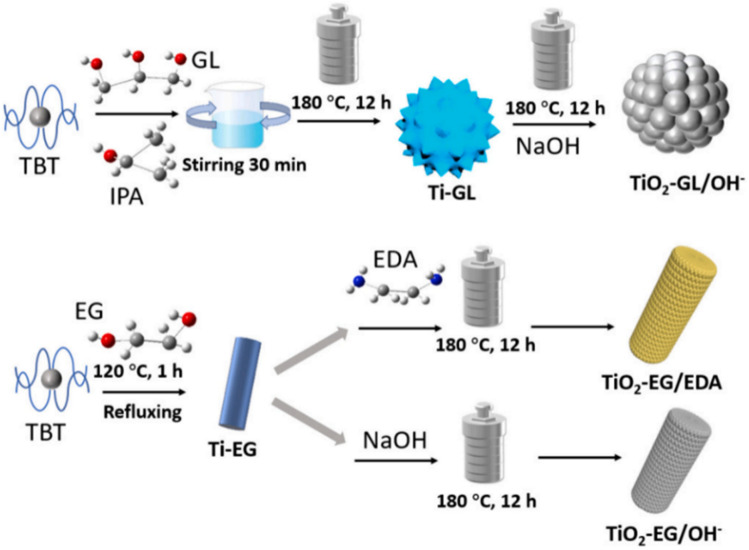
Preparation of the 3 types of TiO_2_ brookite. Reproduced from ref. 53, with permission from Elsevier.

Cheng *et al.*^53^ synthesized three types of TiO_2_ brookite (Fig. 5) by modifying the reaction conditions and tested the degradation capacity of ciprofloxacin (CIP) using these photocatalysts. TiO_2_ brookite is quite under-researched, although it has a suitable band gap for photocatalytic applications, and the recombination rate of the electron–hole pairs in brookite is lower than that in anatase and rutile TiO_2_. Tetrabutyl titanate was used as a precursor for the synthesis of the three catalysts, together with ethylene glycol or glycerol as needed, in either an ethylenediamine or sodium hydroxide medium. For testing the photocatalytic activity, a ciprofloxacin solution with a concentration of 20 mg L^−1^ was used, and irradiation was performed using a 300-W xenon lamp for 3 hours. To achieve adsorption–desorption equilibrium, the samples of ciprofloxacin and the catalyst were initially placed in the dark for 1 hour under stirring. Among the three types of catalysts, the catalyst that was obtained using tetrabutyl titanate and ethylene glycol as precursors in an ethylenediamine medium (TiO_2_-EG/EDA) had the best adsorption capacity due to its larger specific surface area and smaller pore size. It was found that TiO_2_-EG/EDA had the longest electron–hole pair lifetime, the highest electron transport speed, and the most active sites, which were the reasons why the degradation capacity of this catalyst was superior to the other two catalysts. High-resolution mass spectrometry (HRMS) was used to analyze the ciprofloxacin solutions subjected to irradiation for 1, 2 and 3 hours in order to identify ciprofloxacin degradation products. Two ciprofloxacin degradation pathways were suggested based on HRMS data; in both scenarios, CO_2_ and H_2_O were the final products. Tests on the acute toxicity of ciprofloxacin and its degradation products on fish revealed that while ciprofloxacin was not harmful, certain intermediate degradation products (that were rapidly degraded) may be harmful to fish, but the final degradation products did not pose a risk to aquatic organisms.^53^

### 4.3 TiO_2_-based catalysts deposited on different supports

Table 4 summarizes some photocatalysts based on TiO_2_ and deposited on different supports. The table includes information related to the synthesis of the catalysts, their degradation capability for specific antibiotics under specific conditions (dosage and light), and their reusability potential.

**Table 4:** List of reported TiO_2_-derived catalysts and their degradation capacity and reusability under specific conditions

Catalyst	Method	Pollutant	Dosage	Light	Degradation (%)/time	Reusability	Ref.
MXene-derived TiO_2_-supported SiO_2_/Ti_3_C_2_ composite	Ultrasonic and wet impregnation techniques	Tetracycline hydrochloride (THC)	800 mg per L catalyst; 40 mg per L THC	Visible light	95%/80 minutes	5	54
Ag/TiO_2_/coffee ground-derived activated carbon support	Hydrothermal process and photodeposition method	Tetracycline (TC)	—	300-W xenon lamp	99% TC/40 minutes	4	55
		Norfloxacin (NOR)			96% NOR/50 minutes		
Bifurcated dendritic SrCO_3_–TiO_2_/CdS heterostructure	Hydrothermal method	Oxytetracycline (OTC)	15 mg per L OTC	300-W xenon lamp	95.8%/120 minutes	—	56
CuO–TiO_2_ nanocomposite	CuO–TiO_2_ nanocomposites synthesized using a *C. benghalensis* extract	Ciprofloxacin (CIP)	10 mg per L CIP/SSX	UV irradiation	93.64% SSX	—	57
		Sulfisoxazole (SSX)			67.30% CIP		
					120 minutes		
TiO_2_–arabic gum	Sol–gel method	Ciprofloxacin (CIP)	1000 mg per L catalyst; 125 mg per L CIP	160-W mercury vapor lamp	94%/150 minutes	3	58
Cu(ii) porphyrin-sensitized mesoporous anatase	Solvothermal method	Tetracycline (TC)	100 mg per L catalyst; 20 mg per L tetracycline (TC)	300-W xenon lamp	a75%/100 minutes	4	59
Mesoporous TiO_2_–ZnO	Sol-precipitation method	Doxycycline (DOX)	1000 mg per L catalyst; 50 mg per L DOX; pH = 4.4	UV irradiation	100%/30 minutes	10	60
Terbium and manganese coprecipitated TiO_2_	Liquid-phase plasma method	Tetracycline (TC)	200 mg per L catalyst; 10 mg per L TC	LED light	55.87%/180 minutes	5	61
TiO_2_ nanocomposites incorporated with carbon quantum dots (CQDs)	TiO_2_ nanocomposite-incorporated CQDs derived from artificial and natural sources	Tetracycline (TC)	200 mg per L catalyst; 20 mg per L TC; pH = 8	Xenon lamp	91.5%/120 minutes	3	62

a Estimated from the graphics provided by the authors.^59^ The degradation efficiency was not mentioned in the text of the original article.

**Fig. 6:**
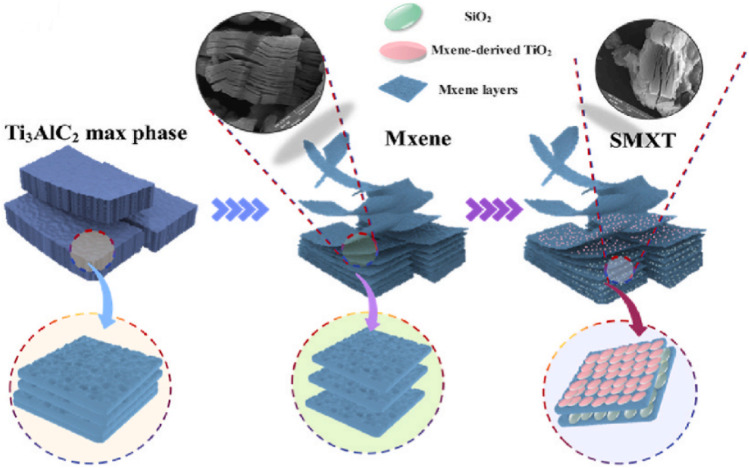
Synthesis of SMXT. Reproduced from ref. 54, based on CC BY license.

Mousavi *et al.*^54^ synthesized an MXene-derived TiO_2_-supported SiO_2_/Ti_3_C_2_ composite (SMXT) by employing ultrasonic and wet impregnation techniques (Fig. 6). The XRD spectrum showed that the aluminum atomic layer in Ti_3_AlC_2_ was completely removed, and Ti_3_C_2_ was successfully prepared. The N_2_ adsorption/desorption isotherm showed that the average diameter of the pores was 4 nm, with a pore size distribution ranging from 2 to 136 nm. Using SiO_2_ as a support material, TiO_2_'s dispersion, structural stability and agglomeration resistance were greatly increased, improving durability and charge separation. The MXene-derived TiO_2_-supported SiO_2_/Ti_3_C_2_ composite demonstrated ideal photocatalytic performance after being annealed at 450 °C because of its specific band gap and decreased electron–hole recombination. The composite achieved 95% tetracycline hydrochloride degradation in 80 minutes under ideal conditions. Additionally, the material demonstrated outstanding recyclability, retaining high efficiency across five consecutive cycles. There are two distinct degradation pathways for tetracycline hydrochloride: a more aggressive pathway that involves quick fragmentation of the aromatic rings and a gentler pathway that involves milder oxidation and the generation of bulkier intermediates. At the end of the degradation process, CO_2_ and H_2_O are obtained in both situations. Tetracycline hydrochloride and some of its intermediate degradation products are thought to be very toxic, but the degradation products with smaller molecules that are obtained towards the end of the irradiation period are thought to be harmless.^54^

**Fig. 7:**
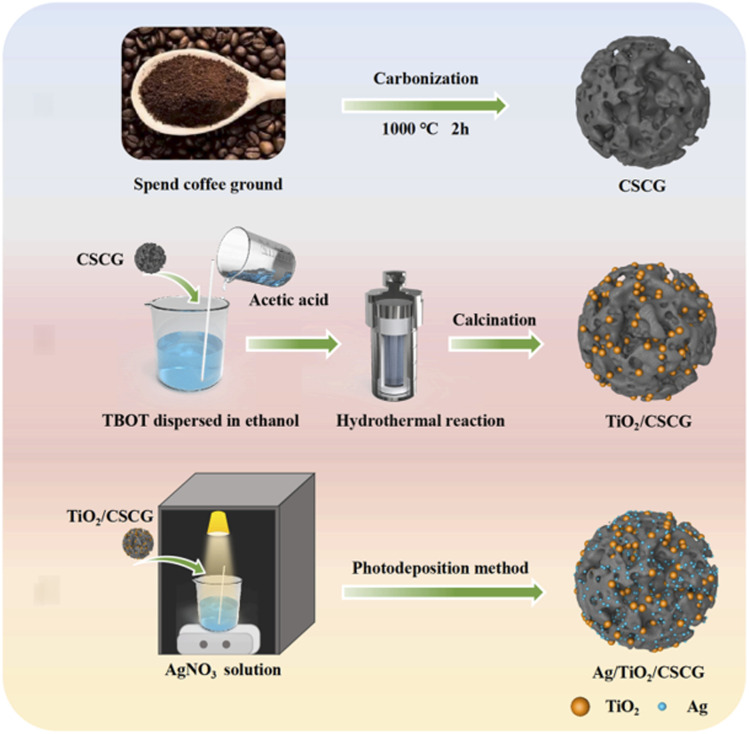
Schematic of the fabrication process. Reproduced from ref. 55, with permission from Elsevier.

Although anatase TiO_2_ is a frequently used catalyst in the photocatalytic degradation of emerging pollutants, the photocatalytic activity of this catalyst is reduced by its broad band gap, particle aggregation tendency, and rapid electron–hole pair recombination. Considering these aspects, Jin *et al.* synthesized a ternary catalyst by growing TiO_2_ and Ag nanoparticles on a used-coffee-ground-derived activated carbon support (Fig. 7).^55^ The photocatalytic activity was tested by the degradation of methylene blue, methyl orange, tetracycline and norfloxacin solutions under a 300-W xenon lamp. In the cases of tetracycline and norfloxacin, the intensity of the characteristic peaks decreased proportionally with the irradiation time, achieving a degradation efficiency of 99% in 40 minutes for tetracycline and 96% in 50 minutes for norfloxacin. Four testing cycles were used to evaluate the synthesized ternary catalyst's reusability, with the photocatalyst being regenerated prior to each new testing cycle. Even after four testing-regeneration cycles, the degradation percentage remained high, indicating an elevated level of recyclability.^55^

**Fig. 8:**
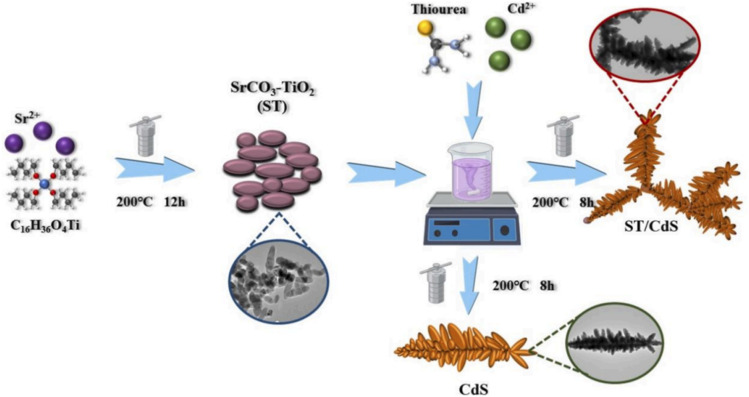
Preparation of the ST/CdS heterostructure. Reproduced from ref. 56, with permission from Elsevier.

Sun *et al.*^56^ developed a bifurcated dendritic heterostructure, SrCO_3_–TiO_2_/CdS (ST/CdS), that could degrade oxytetracycline from wastewater (Fig. 8). Three SrCO_3_–TiO_2_ composites with different Ti : Sr molar ratios were prepared by a facile one-step hydrothermal method, and three SrCO_3_–TiO_2_/CdS heterostructures were prepared by an *in situ* hydrothermal method using different SrCO_3_–TiO_2_ wt%. The assessment of the photocatalytic activity was carried out using a 15 mg per L oxytetracycline solution under 300-W xenon lamp irradiation. To ensure that the adsorption–desorption equilibrium was achieved, the tested solutions were placed in the dark for half an hour before the effective irradiation. The 9ST-2/CdS heterostructure had the highest photocatalytic degradation rate, with a degradation efficiency of 95.8%. Two degradation pathways of oxytetracycline were described. These pathways involved hydroxylation, dihydroxylation, dehydroxylation, deamidation, demethylation, reduction, hydrogenation, dehydration, deamination, decarboxylation, and ring-opening reactions. In both cases, the complete mineralization of oxytetracycline occurred, resulting in the formation of CO_2_ and H_2_O.^56^

Bopape *et al.*^57^ used a *C. benghalensis* extract as a precursor to synthesize TiO_2_ nanoparticles, CuO nanoparticles and CuO–TiO_2_ nanocomposites. The latter was synthesized at three different Cu(NO_3_)_2_·3H_2_O : TiF_4_ ratios, namely, 70 : 30, 50 : 50 and 30 : 70. The degradation capacity was tested against ciprofloxacin and sulfisoxazole (10 mg per L solutions) under UV light irradiation. The highest degradation capacity, 93.64%, was noted for sulfisoxazole by the nanocomposite with a Cu(NO_3_)_2_·3H_2_O : TiF_4_ ratio of 30 : 70, whereas the maximum ciprofloxacin degradation capacity achieved was 67.3% using the nanocomposite with a Cu(NO_3_)_2_·3H_2_O : TiF_4_ ratio of 70 : 30. Four degradation cycles were carried out under conditions comparable to those used in standard photocatalytic degradation experiments in order to ascertain the stability and recyclability of the CuO–TiO_2_ nanocomposites. Following each cycle, distilled water was used to completely wash the photocatalyst, and it was recovered using membrane filtration. After that, the material was dried at 60 °C to produce a powder, which was used in the following cycle. The results of the experiments showed that the nanocomposites were not reusable.^57^

**Fig. 9:**
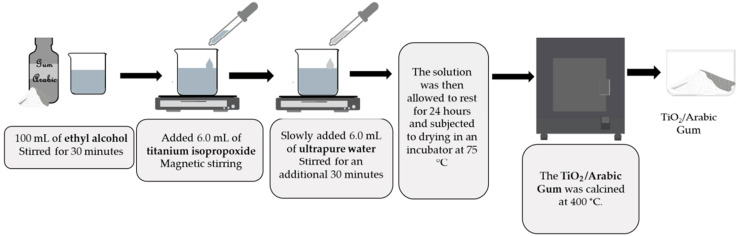
Synthesis of the TiO_2_–arabic gum catalyst. Reproduced from ref. 58, based on the CC BY license.

Lopes *et al.*^58^ synthesized a TiO_2_–arabic gum catalyst capable of photocatalytically degrading ciprofloxacin under UV irradiation (Fig. 9). The TiO_2_–arabic gum catalyst was synthesized using a sol–gel method, and the obtained product was calcined at 400 °C.^63^ The photodegradation process was conducted in a radiation box with a borosilicate reactor connected to a thermostatic bath to keep the temperature constant at 25 °C. The UV light source used for the irradiation was a 160-W mercury vapor lamp without a bulb. Before the irradiation, the suspension was stirred in the dark for 1 hour in order to achieve the adsorption–desorption equilibrium. To investigate the photocatalytic activity and identify the most effective catalyst concentration, three different catalyst concentrations were tested. 1000 mg L^−1^ of the TiO_2_/arabic gum catalyst was found to achieve the highest photodegradation degree of 94%. The material's recyclability was assessed, and it was found that the catalyst maintained its characteristics after three cycles of degradation, with the degradation percentage dropping only slightly to 92%.^58^

**Fig. 10:**
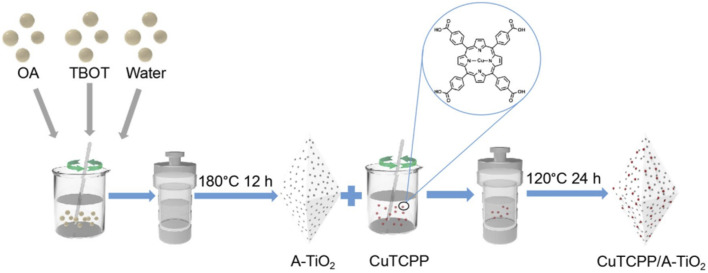
Synthesis of anatase TiO_2_ (A-TiO_2_) and A-TiO_2_ decorated with Cu(ii) porphyrin. Reproduced from ref. 59, with permission from Springer Nature.

Using a solvothermal method, Zhu *et al.*^59^ synthesized mesoporous anatase TiO_2_. To enhance visible light absorption and prevent charge recombination, anatase TiO_2_ was decorated with Cu(ii) porphyrin (Fig. 10). Three photocatalysts with different weight loadings of Cu(ii) 5,10,15,20-tetra(4-carboxylphenyl)porphyrin were reported in this paper. The photocatalytic performance of the synthesized catalysts was evaluated using a 20 mg per L tetracycline solution under visible light irradiation (300-W xenon lamp). Compared to pure anatase TiO_2_, all three synthesized photocatalysts demonstrated superior photocatalytic activity. The catalyst with a 1% weight loading of the Cu(ii) porphyrin derivative was the most efficacious, with an increase in the photocatalytic efficiency of 2.2-fold compared to pure anatase TiO_2_. The photocatalytic efficiency dropped when the loading of the Cu(ii) porphyrin derivative increased over 2.5%, most likely because of the shielding effect. Regarding the reusability of the catalyst, the tests conducted showed that after four testing cycles, there were no significant changes, with only a slight decrease in the tetracycline degradation capacity.^59^

Ani *et al.*^60^ developed a mesoporous TiO_2_–ZnO photocatalyst that was highly effective for doxycycline degradation. Due to its greater absorption of UV light, ZnO has better oxidative properties than TiO_2_. Compared to the photocatalysts based on conventional semiconductors, the synthesized photocatalyst exhibited greater catalytic activity due to the synergy between the two oxides, which enabled it to degrade higher concentrations of emerging contaminants. TiO_2_ was prepared *via* a sol-precipitation method described by Morales *et al.*^64^ ZnO was synthesized using zinc nitrate hexahydrate as a precursor. ZnO and TiO_2_ precipitates in a suspension were combined in a 3 : 1 molar ratio. Different operating parameters, including the concentration of the catalyst, the pH of the testing solution, and the concentration of the doxycycline solution, were tested in order to determine the best conditions to carry out the photocatalytic degradation of doxycycline. The best results were obtained when a 50 mg per L solution of doxycycline was degraded using 1000 mg L^−1^ of the catalyst at a pH of 4.4. Higher catalyst concentrations may have caused aggregation, which prevented the absorption of light radiation. At low doxycycline concentrations (10 mg L^−1^ and 20 mg L^−1^), adsorption was the predominant phenomenon, and photocatalysis may be irrelevant. At basic pH (pH = 11), the double deprotonation of doxycycline occurred, leading to repulsion between OH^−^ in NaOH and the catalyst, resulting in the absence of adsorption. Even after 10 testing cycles, the synthesized photocatalyst maintained its catalytic properties, suggesting that it could be used in industrial applications.^60^

You and Jung^61^ developed a TiO_2_ catalyst coprecipitated with terbium and manganese using a novel method: liquid-phase plasma. The TiO_2_ surface was uniformly precipitated with oxides such as Tb_2_O_3_, TbO_2_, Mn_2_O_3_, and MnO_2_. Among all samples, the band gap energy of TiO_2_ precipitated with manganese decreased the most, and the decrease was greater when TiO_2_ was coprecipitated with Tb/Mn than when terbium was the only precipitant. While coprecipitating with terbium and manganese had a synergistic effect that boosted the photocatalytic efficiency, precipitating with manganese alone lowered the photocatalytic efficiency. The Tb/Mn-TiO_2_ catalyst degraded 56% of the 10 mg per L tetracycline solution within 3 hours under blue LED light irradiation. The photocatalytic efficiency was greater when the amount of the terbium precursor was greater than the amount of manganese. The synthesized photocatalyst was found to retain its photocatalytic efficacy after five degradation cycles. A minor drop in crystallinity was noted, which was explained by the material being cleaned and dried between degradation cycles. The functional groups in the tetracycline structure, such as amine groups, double bonds, and phenolic groups, are easily attacked by the hydroxyl radicals generated in the photocatalysis process. The two proposed degradation pathways led to the production of CO_2_, H_2_O and nitrogen compounds.^61^

**Fig. 11:**
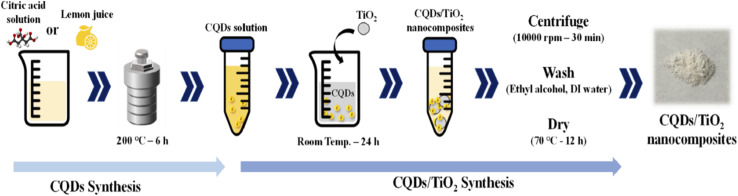
Preparation of CQDs and CQDs/TiO_2_. Reproduced from ref. 62, with permission from Elsevier.

Choi *et al.*^62^ prepared TiO_2_ nanocomposites incorporated with carbon quantum dots for tetracycline removal (Fig. 11). A modified version of the approach proposed by Zhao *et al.* was used to prepare carbon quantum dots (CQDs).^65^ CQDs/TiO_2_ was prepared by mixing TiO_2_ nanoparticles with different amounts of a CQD solution. Lemon juice, a natural precursor, was also used to synthesize CQDs. CQDs/TiO_2_ prepared using natural sources was synthesized under the same conditions as previously mentioned. The testing of photocatalytic activity was carried out in a 20 mg per L tetracycline solution using a Max-350 xenon lamp as the visible light source. Due to the high intensity of radiation in the 420–500 nm region, the radiation source employed during these tests activated TiO_2_, even though TiO_2_ is normally not active in the visible range. CQDs/TiO_2_-4 (with a relative molar ratio of citric acid of 4) exhibited the highest efficiency among the four designed photocatalysts, removing 91.5% of tetracycline under ideal conditions (catalyst dose = 200 mg L^−1^, tetracycline solution = 20 mg L^−1^, solution pH = 8, irradiation period = 120 minutes). Furthermore, it was discovered that overloading TiO_2_ with CQDs reduced the catalytic activity, most likely as a result of a reduction in the number of active sites. Although a decrease in the tetracycline removal capacity was observed after three cycles, the catalytic activity remained high, as confirmed by TEM and X-ray photoelectron spectroscopy (XPS) analysis. There are three possible mechanisms for the degradation of tetracycline: attack on the acylamino group, oxidation of tetracycline molecules, and attack on the aromatic ring of tetracycline. If the irradiation period is extended, the intermediate degradation products may mineralize to CO_2_ and H_2_O.^62^

### 4.4 Titanate-based catalysts

Cerón-Urbano *et al.* prepared CaTiO_3_ mesoporous nanoparticles using the Pechini polymeric precursor method at 700 °C for 6 hours.^66^ The photocatalytic activity was tested in a photodegradation chamber equipped with an 800-W mercury lamp. The initial concentration of the levofloxacin solution was 10 mg L^−1^, and the solution's pH was set at 4.5. The degradation tests were carried out at four different catalyst doses, namely, 3000, 5000, 10 000 and 15 000 mg L^−1^. In the absence of light irradiation, CaTiO_3_ nanoparticles did not absorb antibiotic molecules. The levofloxacin solution significantly degraded when exposed to ultraviolet light in the presence of the nanoparticle catalyst. The best photocatalytic activity was achieved at a 10 000 mg per L catalyst concentration, which was 98.1% after 3 hours of irradiation. The percentage of levofloxacin degradation increased when the catalyst dose was increased from 3000 to 10 000 mg L^−1^, while the degradation efficiency declined when the CaTiO_3_ dose was increased to 15 000 mg L^−1^.^66^

**Fig. 12:**
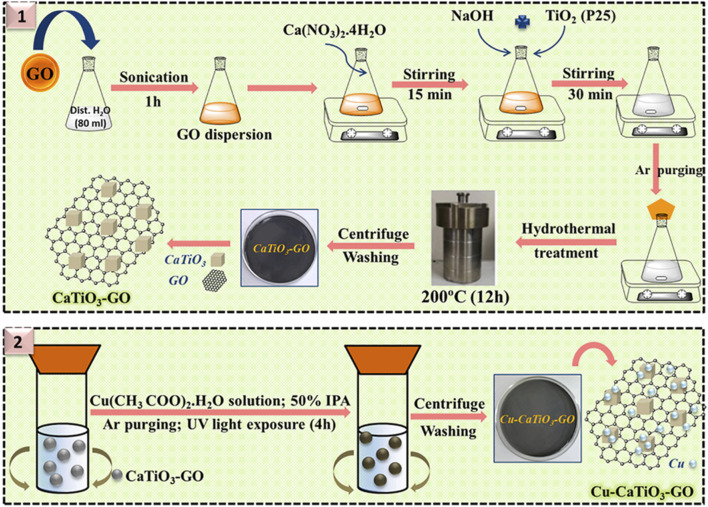
Preparation process of the binary composite CaTiO_3_-GO (1) and the ternary composite Cu-CaTiO_3_-GO (2). Reproduced from ref. 67, with permission from Elsevier.

Passi and Pal synthesized the ternary catalyst Cu-CaTiO_3_-GO through the photodeposition of Cu nanoparticles over a CaTiO_3_-GO composite (GO = graphene oxide) (Fig. 12).^67^ The assessment of the photocatalytic activity was completed under visible LED light irradiation using 10 mL of a 50 mg per L cefixime solution and 10 mg of the catalyst. The ternary photocatalyst exhibited the highest degradation percentage, 94.1% after 100 minutes of irradiation. Pure CaTiO_3_ exhibited the lowest photocatalytic activity, which may be explained by its large band gap, poor light harvesting capacity, increased charge carrier recombination and reduced surface area. Compared to Cu-CaTiO_3_, CaTiO_3_-GO demonstrated superior catalytic activity. Regarding the reusability of the ternary composite, the degradation efficiency decreased after four consecutive degradation cycles, but the catalyst continued to show 79.4% removal efficiency for cefixime. The decrease in its removal efficiency may be the result of catalyst loss during the retrieval procedure. Three pathways for the degradation of cefixime were proposed; pathways that, through prolonged exposure to visible light, led to the formation of simple inorganic compounds: CO_2_, H_2_O, ammonium and sulfate ions.^67^

The gap between laboratory and field implementation is a major concern and needs to be considered. Unfortunately, most of the tests are performed in idealized, laboratory-scale conditions, without considering the conditions of unpredictable ionic strength and high levels of other contaminants (which can consume the reactive oxygen species), and thus, scale-up is needed to check the efficiency of the prepared catalysts. To overcome the risks of inactivation or blocking of the activity because of competing reactions with other components from real solutions (such as dyes, organic wastes, and other materials with low toxicity), supported catalysts (such as mesoporous supports or even molecularly imprinted materials) that allow only specific antibiotics to reach the active sites to increase the degradation efficiency should be considered. However, other supports can be used, such as C-based materials, which are able to adsorb specific pollutants, and the developed photocatalysts should generate the reactive species (which are concentrated on their surface) that efficiently degrade these pollutants.

Considering the multiple examples presented above, it is obvious that mixed-metal oxide/titanate heterostructures could be the solution to drastically improve charge separation and redox potential, broaden photoresponse, and assure synergy.^70^

Considering the AI age we are living in, new approaches benefiting from the AI's prediction power in materials (photocatalysts) development have to be considered.^71,72^ Certainly, this means that there is a need to develop new AI-based supervised learning models, including support vector machines (SVMs), artificial neural networks (ANNs), and tree-based algorithms. Using such tools, research will be better focused on highly probable solutions, thereby proposing material combinations that yield the highest activity, selectivity, and stability and significantly shortening the innovation cycle in a more sustainable way by consuming less chemicals and resources.

## 5. Toxicity of the degradation by-products

The toxicity and potential of the degradation products to cause antibiotic resistance are not well understood. Degradation leads to several products and ratios; so, the overall toxicity is dependent on each component and its concentration. The potential of these molecules to induce antibiotic resistance is just marginally considered in the literature, but several studies have also addressed aspects regarding the toxicity of the compounds generated during irradiation. Moreover, the overall toxicity and capacity to induce resistance are very complex because many other aspects have to be considered, including, but not limited to, the presence of other stressors such as pesticides and heavy metals. In Table 5, three commonly present antibiotics are listed, along with their oxidative degradation pathway. It is quite clear that in each case, tens of products are released, their ratio varies according to the conditions, and, more importantly, the toxicity of these products varies as well. Unfortunately, there are still limited data in the literature related to the influence of these compounds in generating resistance.

**Table 5:** Degradation pathways of some relevant antibiotics

Antibiotic	Degradation pathway	Toxicity assessments; ref.
Tetracycline	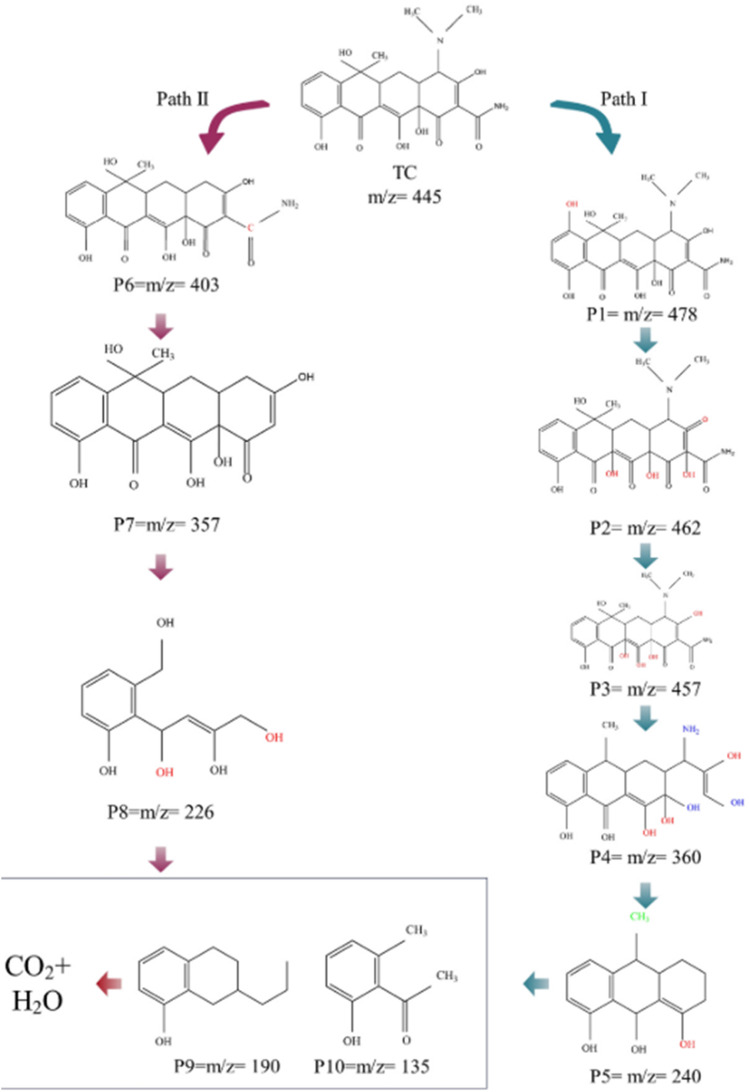	Products P1, P2, P3, and P6 are considered to be “very toxic”
		Products P4, P5, P7, P8, P9, and P10 are classified as “not harmful”
		Reproduced from ref. 54, based on CC BY license
Oxytetracycline	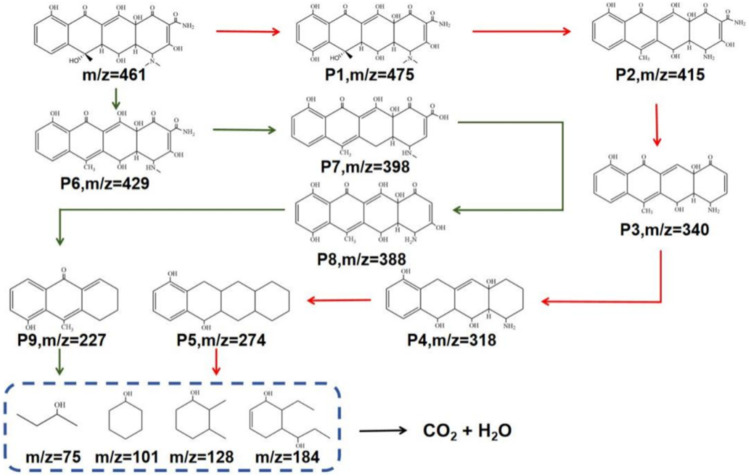	The majority of the degradation products show higher toxicity than oxytetracycline^56,68^
		Reproduced from ref. 56, with the kind permission of Elsevier
Cefixime	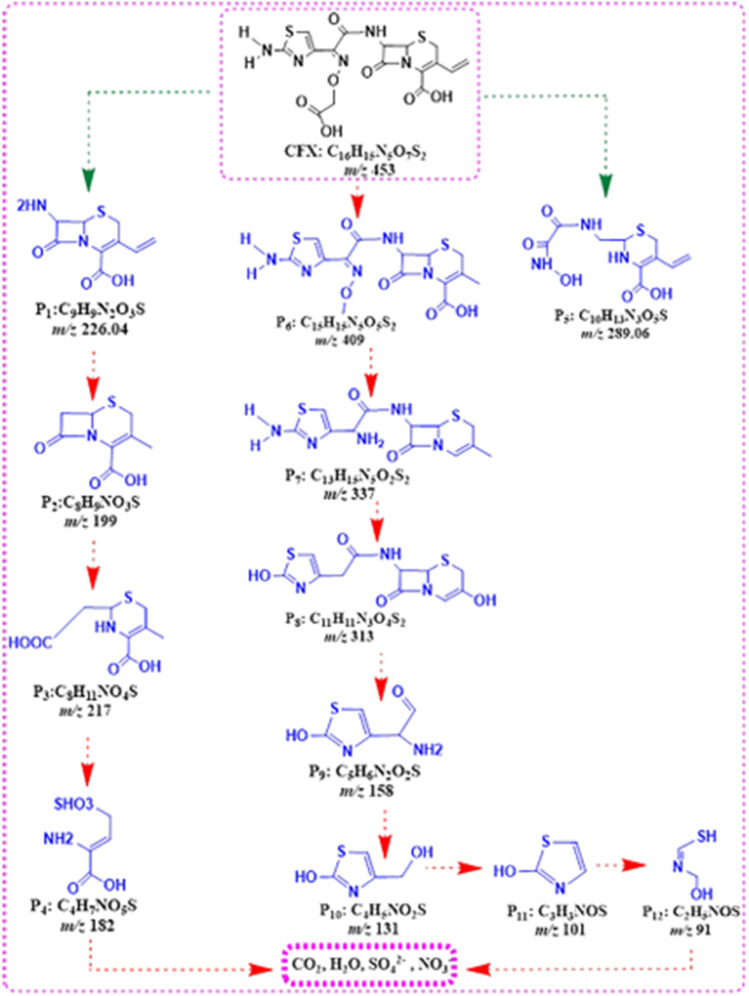	There are no conclusive data on the toxicity of cefixime degradation by-products, but ceftiofur, another cephalosporin antibiotic, generates a more toxic compound upon degradation than the parent compound^67,69^
		Reproduced from ref. 67, with permission from Elsevier

A major goal of these studies is the development of solutions that can assure complete and safe mineralization. This means that suitable techniques, such as advanced oxidation processes, have to be developed to completely transform antibiotics into the simplest oxides or derivatives (CO_2_, H_2_O, NO_2_^−^, NO_3_^−^, SO_3_^2−^, SO_4_^2−^, *etc.*) that have well-known toxicity and can be easily handled by the nature. Certainly, if this is not possible, the ecological structure–activity relationships (ECOSAR) model can be used as a valuable computational tool for predicting the toxicity of intermediates and guiding the design of catalysts that promote complete-degradation pathways. Further, to confirm these data, rigorous biological toxicity assays should be performed for validating the environmental safety of the final effluent.^70^

## 6. Conclusions and future perspectives

Due to the pharmaceutical industry's tremendous growth, high microbial loading and virulence, the use of antibiotics has increased over the years. Antibiotic resistance is a global phenomenon that has emerged as a result of several factors, including uncontrolled antibiotic use and (intended or unintended) antibiotic release into nature. It is essential to find alternative methods to eliminate these antibiotics before they are disseminated into the environment and cause the development of resistant genes in wastewater or in the waters in which they are released. The most recent research studies on the application of titanium-based nanostructures for the photocatalytic degradation of different antibiotics were highlighted in this review. Relatively few studies have been reported on the incorporation of titanium dioxide nanoparticles into the pores of mesoporous silica and the potential application of this binary catalyst for antibiotic photodegradation. This review highlighted the superior photocatalytic efficiency of TiO_2_-based catalysts deposited on different supports with large specific surface areas, as well as titanate-based catalysts; antibiotic removal percentages were typically greater than 90%, and the catalytic structures typically retained their efficiency for at least three photodegradation cycles, assuring circularity and sustainability. Furthermore, not many studies have reported on the toxicity of degradation products, which is crucial given that the reaction products need to be nontoxic or at least less toxic than the parent compound in order to demonstrate that the antibiotic was effectively degraded. Another important aspect that has to be carefully assessed in the future is related to the evaluation of each component resulting from the degradation process to assess its ability to generate resistance within the specific bacterial community.

## Conflicts of interest

There are no conflicts to declare.

## Data Availability

No primary research results, software or code have been included, and no new data were generated or analysed as part of this review.
